# The Value of Continuous Electrocardiographic Monitoring in Pediatric Cardiology: A Local Center Experience

**DOI:** 10.7759/cureus.25667

**Published:** 2022-06-05

**Authors:** Areej Alotaibi, Ghadir A Alakhfash, Ali Alakhfash, Tayseer Mahmoud, Alhasan A Alakhfash, Abdullah Al Qwaee, Abdulrahman Mesned

**Affiliations:** 1 Department of Pediatric Cardiology, Prince Sultan Cardiac Center - Qassim, Buraydah, SAU; 2 Pediatric Cardiology, Sulaiman Al Rajhi University, Buraydah, SAU; 3 Cardiovascular Sciences, Sapienza Università di Roma, Rome, ITA; 4 Pediatrics, Sulaiman Al Rajhi University, Buraydah, SAU

**Keywords:** congenital heart diseases (chds), pediatric electrophysiology, holter monitoring, sudden cardiac death, pediatric arrhythmias

## Abstract

Objectives

This study aims to evaluate the value of Holter monitoring in pediatric cases and look for the best predictor for abnormal Holter monitoring.

Methodology

All patients referred with cardiac symptoms associated or possibly related to abnormal cardiac rhythm from January 2019 to December 2020 were retrospectively reviewed. The demographic, clinical, 12-lead electrocardiography (ECG), echocardiography, and Holter monitoring results were reviewed. Multinomial logistic regression analysis was used to assess the correlation between gender, age, type of symptoms, ECG, and echo abnormalities, and Holter monitoring results were analyzed.

Results

During the study period, a total of 189 Holter monitoring was performed for 187 patients. The mean age at the performance of Holter monitoring was 88.6 ± 57 months. The female/male ratio was 1.5:1. The commonest indications for Holter monitoring were abnormal 12-lead ECG (30.7%), palpitations (30.7%), syncopal attacks (12.7%), and chest pain (6.9%). Patients with congenital heart disease (CHD) pre- or post-cardiac intervention constitute 9% of the total Holter monitoring cases. Apart from sinus arrhythmia, 12-lead ECG was abnormal in 57 (30%) patients, with premature atrial complexes (PACs) being the most common abnormality. Echocardiography was abnormal in 67 (35.4%) cases, with secundum atrial septal defect (ASD) (6.3%) and mitral valve prolapse (5.8%) being the commonest abnormalities. The Holter monitoring was completely normal in 89 (47.1%) cases. The commonest Holter abnormalities were PACs (12.7%), supraventricular tachycardia (SVT) (5.8%), and premature ventricular complexes (PVCs) (4.8%). There were 24 patients with SVT, and eight of them had normal Holter monitoring. One patient with SVT had ablation by the electrophysiologist. Using the multinomial logistic regression analysis, significantly abnormal 12-lead ECG, the presence of CHD, and abnormal echocardiography predict the presence of abnormal Holter results with a statistically significant p-value.

Conclusion

Most pediatric arrhythmias are benign. Holter monitoring provides reassurance for the patient and family. Abnormal Holter monitoring is more often observed in patients with paroxysmal or persistently abnormal 12-lead ECG with or without associated cardiac abnormalities or cardiac interventions. The yield of Holter monitoring is low in children referred because of chest pain, palpitations, or syncope with no other cardiac symptoms and with a structurally and functionally normal heart.

## Introduction

Although most childhood arrhythmias are benign, a prompt and correct diagnosis of a serious rhythm disturbance in a child can be lifesaving [1]. Several tools are available to document arrhythmias in the workup of a patient with palpitation, including 24-hour Holter monitoring, 30-day external continuous monitoring, and implantable loop recorders [2].

The Holter monitor is a small, portable, noninvasive ambulatory diagnostic tool used for continuously recording the electrical activity of the heart over a 24- to 72-hour period. It is sometimes referred to as an “ambulatory electrocardiography” or “ambulatory ECG.” It was first introduced by the American biophysicist Norman J. Holter (1914-1983) in the 1940s [3].

Advances in technology allow remote monitoring of heart rhythms through a wide variety of devices, including ambulatory external monitors, implantable event recorders, pacemakers, and cardioverter-defibrillators [4].

Cardiac Holter monitors can provide information about the correlation between the patients’ symptoms and electrocardiographic activities, asymptomatic arrhythmias, type of arrhythmia and how long it lasts, possible arrhythmia triggers, and the effectiveness of antiarrhythmic medications [5].

The value of Holter monitoring depends on capturing episodes of abnormal cardiac rhythm that can happen during the recording time (24 hours or longer). Even in the presence of significant arrhythmia, the rhythm abnormality might not happen during the recording period [6].

In some cases, in the presence of symptoms attributed by the patient and/or family to cardiac disease, in the presence of normal ECG and echocardiography, Holter monitoring might add to the reassurance and relief of the patients’ anxiety about the presence of significant cardiac abnormality [2].

This study aims to evaluate the value of Holter monitoring in pediatric cases and look for the best predictor for abnormal Holter monitoring.

## Materials and methods

A retrospective study was conducted in the pediatric cardiology departments at Prince Sultan Cardiac Center (PSCC) - Qassim, Maternity and Children Hospital (MCH), Buraydah. The department database, patient’s progress notes, ECGs, echocardiography, and Holter reports were reviewed. All patients who had 24-hour Holter monitoring in the pediatric cardiology department from January 2019 to December 2020 were included. The demographic data and the indication for referral to pediatric cardiology and for Holter monitoring were reviewed. The 12-lead ECG findings, either from the referring hospital or the one performed in the pediatric cardiology clinic, were reviewed. The patient clinical and family history, physical examination, 12-lead ECG recordings, and echocardiographic findings were reviewed. The 24-hour Holter ECG recordings were reviewed by an expert pediatric cardiologist, and if any abnormal findings were found, the Holter monitor will be reviewed also by a pediatric electrophysiologist.

The study was approved by the institutional research committee on May 30, 2021 (letter number: 21-1019).

The patients were categorized according to the age group, reason for referral and Holter monitoring, and presence or absence of ECG and echocardiography abnormalities.

Normal ECG is defined based on the recommendations for normal ECG for age and gender. Abnormal Holter findings were defined based on the presence of either abnormal rhythm and/or abnormal heart rate. Holter abnormalities were divided into either significant abnormalities requiring follow-up and/or medication or intervention or insignificant abnormalities such as rare or occasional premature atrial complexes (PACs) or premature ventricular complexes (PVCs), and transient, non-sustained first- or second-degree heart block. Abnormal Holter findings were considered significant in any patient with congenital heart disease (CHD) or who had cardiac surgery or catheterization.

The correlation between the Holter findings and the different variables, mainly the demographic, clinical, referral causes, and 12-lead ECG findings, was created using the multinomial logistic regression analysis. The chi-squared test was used to test the significance with a 95% confidence interval. A p-value of 0.05 or less was considered significant. The SPSS Statistics version 25.0 (IBM Corp., Armonk, NY, USA) was used for statistical analysis. Numerical variables were presented as mean ± standard deviation (SD) and median with minimum and maximum results. Categorical variables were represented as numbers and percentages. The study was approved by the institutional research committee.

Frequent PVCs were defined as ≥5% on 24-hour Holter recording. A wandering pacemaker is used when the ECG shows an irregular rhythm with ongoing changes in P wave morphology, with associated changes in PP interval during more than two beats. The other types of arrhythmias were categorized according to the published definition and classification [7].

Technical considerations

A Holter monitor can be attached to a child of any age, based on the advice of the treating cardiologist. Before Holter monitoring, all patients are evaluated completely, including history, physical examination, 12-lead ECG, and echocardiography. The length of time of Holter monitoring was determined by the treating cardiologist; most of the time, the recording was for 24 hours. A cardiac technician attaches and removes the Holter monitor. Holter monitors may be attached within an inpatient or outpatient setting. While the Holter monitor is attached, the child is allowed to have normal activities, apart from getting the Holter monitor wet and avoiding electric and magnet signals that may affect the recording. The patient/parent/caregiver is encouraged to record activities and any symptoms in the Event’s Card.

## Results

During the study period, the total number of 24-hour Holter monitoring performed for 187 patients was 189. Two patients with supraventricular tachycardia (SVT) and atrioventricular reentry tachycardia (AVRT) had repeated 24-hour Holter monitoring during the study period. They were on medications, and Holter monitoring was done during follow-up. The mean age at the performance of Holter monitoring was 88.6 ± 57 months (median: 100.2 months, mode: 132 months). The female/male ratio was 1.5:1 (N = 113 (60% females and 40% males)). The commonest indications for referral to pediatric cardiology and performance of Holter monitoring were abnormal ECG, palpitations, chest pain, and syncopal attacks (Table 1).

**Table 1:** Indications for referral to pediatric cardiology and Holter monitoring in descending order CHD: congenital heart disease

Indication	Frequency	Percentage (%)
Palpitations	58	30.7
Abnormal 12-lead ECG	58	30.7
Syncope	24	12.7
Chest pain	13	6.9
Arrhythmia post-cardiac intervention	13	6.9
CHD with arrhythmia	7	3.7
Exercise intolerance	6	3.2
Seizure disorder	4	2.1
Fetal arrhythmia	3	1.6
Cardiomyopathy	3	1.6
Total	189	100

Patients with CHDs pre- or post-cardiac intervention constitute 9% of the total Holter monitoring cases. Apart from sinus arrhythmia, 12-lead ECG was abnormal in 57 (30%) patients, with premature atrial complexes being the most common abnormality (Table 2).

**Table 2:** ECG abnormalities in referred patients EAT: ectopic atrial tachycardia, PACs: premature atrial complexes, PPM: permanent pacemaker, PVCs: premature ventricular complexes, RBBB: right bundle branch block, SVT: supraventricular tachycardia

ECG abnormality	Frequency	Percentage (%)
Normal	131	69.31
Ectopic atrial rhythm	9	4.76
Preexcitation	7	3.70
Complete heart block	5	2.65
PACs	5	2.65
PACs, PVCs	5	2.65
First-degree heart block	3	1.59
PVCs	4	2.12
Second-degree heart block	3	1.59
PPM	3	1.59
Sinus bradycardia	3	1.59
EAT	2	1.06
RBBB	2	1.06
Sinus bradycardia	2	1.06
Abnormal Q waves	1	0.53
Ectopic atrial rhythm	1	0.53
PACs	1	0.53
Sinus tachycardia	1	0.53
SVT	1	0.53
Total	189	100

Echocardiography was abnormal in 67 (35.4%) cases, with the commonest type of discovered CHD in the referred patients being ASD secundum and mitral valve prolapse. About 12.6% (24) of the patients who had Holter monitoring underwent cardiac surgery or cardiac catheterization.

The Holter monitoring was completely normal in 92 (48.7%) cases. The commonest Holter abnormalities were PACs, PVCs, and SVT (Table 3). The mean ± SD, minimum and maximum, and average heart rates during the 24-hour Holter monitoring were 61 ± 16, 166 ± 35, and 97 ± 48 beats per minute. Holter monitoring was performed in all age groups but most commonly in those between six and 14 years of age (a total of 117 cases (62%)). Significantly abnormal Holter findings were more common in those with abnormal 12-lead ECG findings either from the referring hospital or the one performed during clinic evaluation. In our study, the presence of transient or persistently abnormal 12-lead ECG was associated with abnormal Holter. Abnormal ECG was present in 87 (46%) of our patients either in the ECG performed at the initial symptoms or the ECG performed in the clinic or inpatients. Among them, 31 had significantly abnormal Holter findings, and 28 had minor, insignificant abnormal Holter findings. Among all cases, 38 (20.1%) patients had abnormal ECG on clinic evaluation, and 23 of them had significant findings on Holter monitoring. The commonest ECG abnormality was SVT, PACs, and PVCs. Abnormal 12-lead ECG, the presence of CHD, and abnormal echocardiography predict the presence of abnormal Holter results with a statistically significant p-value (Table 4).

**Table 3:** Main 24-hour Holter monitoring findings EAT: ectopic atrial tachycardia, PACs: premature atrial complexes, PPM: permanent pacemaker, PVCs: premature ventricular complexes, RBBB: right bundle branch block, SVT: supraventricular tachycardia, WPW: Wolff-Parkinson-White syndrome

Main Holter finding	Frequency	Percentage (%)
Normal	89	47.1
Occasional PACs	24	12.7
SVT	11	5.8
Frequent PVCs	9	4.8
Occasional PVCs	9	4.8
Wandering atrial pacemaker	6	3.2
CHB with junctional escape	5	2.6
EAT	5	2.6
Second-degree heart block	4	2.1
WPW, no SVT recorded	4	2.1
PPM	3	1.6
Frequent PACs, occasional PVCs	2	1.1
Frequent PACs	2	1.1
Junctional rhythm	2	1.1
Occasional PACs and PVCs	2	1.1
Occasional second-degree HB	2	1.1
First-degree heart block	1	0.5
Frequent PACs and PVCs	1	0.5
Intermittent WPW	1	0.5
Occasional PVCs	1	0.5
Occasional PACs	1	0.5
Occasional PACs	1	0.5
Occasional first-degree HB	1	0.5
Occasional PACs, prolonged QT	1	0.5
Occasional sinoatrial block	1	0.5
RBBB	1	0.5
Total	189	100

**Table 4:** Cross-tabulation comparison between those with normal and significantly abnormal Holter monitoring results AVRT: atrioventricular reentry tachycardia, CHB: complete heart block, CHD: congenital heart disease, CMP: cardiomyopathy, DORV: double outlet right ventricle, EAT: ectopic atrial tachycardia, ECG: electrocardiogram, ED: emergency department, LV: left ventricle, MVP: mitral valve prolapse, PACs: premature atrial complexes, PS: pulmonary valve stenosis, PVCs: premature ventricular complexes, SCD: sudden cardiac death, SVT: supraventricular tachycardia, TOF: tetralogy of Fallot, VSD: ventricular septal defect, WPW: Wolff-Parkinson-White syndrome

Variable		Main Holter findings (significant or not significant)			Total	P-value
		Normal	Significant	Not significant		
Gender	Female	55	26	33	114	0.924
	Male	35	19	21	75	
Age group	One month or less	4	11	7	22	0.016
	>1 month-1year	6	6	4	16	
	1-3 years	6	4	1	11	
	>3-6 years	11	4	8	23	
	>6-13 years	38	10	25	73	
	>13 years	25	10	9	44	
CHD	No	63	36	27	126	0.005
	Yes	27	9	27	63	
Cardiac surgery	No	82	41	43	166	0.112
	Yes	8	4	11	23	
Interventional cardiac catheterization	No	82	44	42	168	0.004
	Yes	8	1	12	21	
History of palpitation	No	44	31	32	107	0.075
	Yes	46	14	22	82	
History of syncope	No	75	38	50	163	0.237
	Yes	15	7	4	26	
History of chest pain	No	65	40	42	147	0.072
	Yes	25	5	12	42	
Family history of SCD	No	88	45	53	186	0.434
	Yes	2	0	1	3	
Family history of arrhythmia	No	90	43	52	185	0.071
	Yes	0	2	2	4	
Family history of CHD	No	88	45	51	184	0.418
	Yes	2	0	3	5	
Examination	Normal	78	39	38	155	0.039
	Abnormal	12	6	16	34	
ECG in clinic	Normal	77	22	36	135	0.001
	Abnormal	12	23	19	54	
Echocardiography	Normal	62	34	26	122	0.009
	Abnormal	28	11	28	67	
Holter normal abnormal	Normal	74	7	12	93	0.001
	Abnormal	15	38	43	96	
Medications	No	78	33	49	160	0.054
	Yes	12	12	5	29	
History of SVT	No	84	29	52	165	0.001
	Yes	6	16	2	24	
History of ablation	No	89	45	54	188	0.475
	Yes	1	0	0	1	
Current condition	Stable	63	22	41	126	0.001
	Discharge	16	1	3	20	
	Follow-up with EP	11	22	10	43	
Total		90	45	54	189	

There were 24 patients with SVT; eight of them had normal Holter monitoring (no attacks of SVT during the 24-hour ECG recordings). One patient with SVT and AVRT was diagnosed by history and 12-lead ECG, with no attacks of SVT during the 24-hour Holter monitoring. This patient was referred to ablation by the electrophysiologist (the only case in our cohort who had ablation).

Patients with significant arrhythmia were referred for follow-up by the electrophysiologist (42 patients (22.8%)). Antiarrhythmic medications were given to 29 (15.3%) patients, mainly propranolol. One patient with AVRT had a successful ablation therapy. All patients are alive with no reported sudden cardiac death among them.

Four of our patients were referred because of seizure disorders to exclude arrhythmia or conduction abnormalities leading to a condition mimicking seizure disorder. All of them had normal 24-hour Holter monitoring.

## Discussion

In patients at high risk for sudden cardiac death (SCD), Holter monitoring can give information about the type of arrhythmia and helps in the risk stratification of patients [1].

There are common variations in rhythm in pediatrics, which may be normal, including sinus arrhythmia, short sinus pauses of <1.8 seconds, first-degree atrioventricular block, Mobitz type 1 second-degree atrioventricular block, junctional rhythm, and ventricular or supraventricular extrasystole [7,8]. Based on these recommendations, the presence of one of these findings was considered minor abnormalities in Holter’s monitoring of our patients, and they were discharged if there is no other indication to continue to follow-up with a pediatric cardiologist.

Sinus arrhythmia, ectopic atrial rhythm, “wandering pacemaker,” and junctional rhythm can be normal characteristics in children (15%-25% of healthy children can have these rhythms on the electrocardiogram) [8].

Holter monitoring can give information about the frequency type and rate of cardiac arrhythmias. Recorded events might include an abnormality in the rate, abnormal rhythm, or conduction abnormality.

Wandering atrial pacemaker rhythm is found in 25% of healthy newborn infants, 34% of healthy 10- to 13-year-old boys, 26% of 14- to 16-year-old boys, and 54% of medical students. Atrial ectopic rhythm is distinguished from wandering atrial pacemaker rhythm by its unchanging P wave axis/morphology [9].

PVCs are frequently documented in children. Frequent PVCs (≥500/24 hours) may be addressed as a benign condition and should not preclude sports participation in asymptomatic children and normal cardiac structure and function [10]. PVCs in children with structurally normal hearts have a relatively benign course, with a trend toward spontaneous resolution [11]. PVCs were one of the commonest recorded events in our patients, with an event rate of 4.8% for frequent PVCs and an additional 4.8% for occasional PVCs. Frequent PVCs were more common in patients with an abnormal baseline 12-lead ECG and/or structural heart disease.

Left ventricular (LV) dysfunction could be the result of frequent PVCs and asymptomatic VTs. The development of LV dysfunction in such cases is associated with a higher burden of PVCs and the presence of VTs. LV dysfunction appears to be reversible if the burden of PVCs is decreased by medication or ablation [12]. During the two years of follow-up, no one of our patients had LV dysfunction as a consequence of PVCs.

Patients with obstructive apnea are at risk for arrhythmia, mainly PVCs, with about 30% of them having abnormal ECG during polysomnogram study, with 8% of them having minor cardiac pathology, including atrial and ventricular ectopy, tuberous sclerosis, mitral regurgitation, and aortic insufficiency [13].

The yield of Holter monitoring in detecting arrhythmias was reported by multiple investigators with a high diagnostic yield in detecting arrhythmias with an extended 48-hour Holter monitoring. The commonest indication for Holter monitoring were palpitations, syncope/pre-syncope, chest pain, shortness of breath, and color change/pallor. Holter recording was positive in 11%-37% of patients. The commonest abnormalities are frequent premature ventricular contractions (11.2%) and atrial ectopic beats (8.4%) [14,15]. In our study, the presence of transient or persistently abnormal 12-lead ECG was associated with abnormal Holter. Abnormal ECG was present in 87 (46%) of our patients either in the ECG performed at the initial symptoms or the ECG performed in the clinic or inpatients. Among them, 31 had significantly abnormal Holter findings, and 28 had minor, insignificant abnormal Holter findings. Abnormal 12-lead ECG, the presence of CHD, and abnormal echocardiography predict the presence of abnormal Holter results with a statistically significant p-value (Table 4).

Chest pain in children is usually benign but can cause anxiety to the child and family, with reported normal ECG in more than 95% of cases referred because of chest pain [16]. In this study, there was no effect of chest pain in the percentage of abnormal Holter results. The commonest Holter abnormalities in children referred to because of chest pain are PACs (five cases), wandering atrial pacemaker (three cases), PVCs, and first-degree heart block (two cases).

Syncope is defined as sudden transient loss of consciousness, followed by spontaneous complete recovery, caused by transient global hypoperfusion of the brain. Most episodes of syncope in pediatrics are benign; 5% could be the initial manifestation of a life-threatening cardiac disease [17]. An abnormal history, physical examination, or electrocardiogram is identified in most patients with a cardiac cause of syncope. In the absence of a positive screen result (abnormal history, P/E, and ECG), echocardiogram is most likely normal in children with syncope [18]. Exercise-induced syncope is highly associated with cardiac origins, mainly with ECG abnormalities with susceptibility to arrhythmia but with normal echocardiography in most cases [19]. In this study, there was no effect of syncope in the percentage of abnormal Holter results. The commonest Holter abnormalities in children referred to because of chest pain are PACs (four cases) and occasional PVCs (two cases).

In addition to 12-lead ECGs, Holter monitoring might be performed for patients with a first seizure disorder to exclude arrhythmias such as heart block, long QT interval, and Brugada syndrome [20]. Four of our patients had seizure disorder with normal ECG and 24-hour Holter monitoring.

In pediatric patients, the optimal duration of emergency department (ED) and post-ED cardiac rhythm monitoring for arrhythmia among patients with syncope are unknown. In adult patients, the Canadian Syncope Risk Score is used to categorize patients with syncope. Studies show that in adult patients, the overall arrhythmia risk and the risk after two hours of ED arrival for Canadian Syncope Risk Score low-risk patients is very low [21].

The predictors for sudden death (SD) in patients’ post-cardiac surgery include mainly the presence of symptoms of arrhythmia and/or heart failure during follow-up and/or a history of documented arrhythmia. Twelve-lead ECG, chest X-ray, and Holter ECG findings were not predictive of SD. Most SD events occur during exercise, with ventricular tachycardia/ventricular fibrillation being the most recorded rhythm abnormalities [22,23]. There was no reported SCD event in our cohort.

In patients with cardiomyopathy, routine Holter screening rarely demonstrates significant findings or changes the management plan. Using logistic regression, patients with cardiomyopathy and a history of ventricular arrhythmia with frequent premature ventricular complexes are at a risk for sudden cardiac death [24]. Holter monitoring is not performed routinely in our clinic for patients with cardiomyopathy unless there are ECG abnormalities (arrhythmias). This could be explained by the practice of close monitoring and follow-up of pediatric cases with cardiomyopathy (CMP) and the frequent performance of 12-lead ECGs with every clinic follow-up.

The limitations of the study include its retrospective nature. There might be patients with unidentified arrhythmias who were not referred to our clinic. Patients with CHDs and those who underwent post-cardiac surgeries are not included as Holter monitoring is not routinely performed for all of them.

## Conclusions

Holter monitoring in children is an inexpensive and noninvasive investigation with reasonable diagnostic yield in detecting arrhythmias. Abnormal Holter monitoring is more often observed in patients with paroxysmal or persistently abnormal 12-lead ECG with or without associated cardiac abnormalities or cardiac intervention. The yield of Holter monitoring is low in children referred because of chest pain, palpitations, or syncope with no other cardiac symptoms and with a structurally and functionally normal heart.
